# Neurodevelopment in Preterm Children at 12 Months: Aligning Clinical Observations and Parental Insight

**DOI:** 10.3390/children12091132

**Published:** 2025-08-27

**Authors:** Barbara Caravale, Antonella Castronovo, Loredana Narducci, Cristina Zitarelli, Elsa Addessi, Michela De Cicco, Gianluca Terrin, Camilla Gizzi

**Affiliations:** 1 Department of Developmental and Social Psychology, Sapienza University of Rome, 00185 Rome, Italy; 2 Neonatal Intensive Care Unit, Sant’Eugenio Hospital, 00144 Rome, Italycamilla.gizzi@aslroma2.it (C.G.); 3 Unit of Cognitive Primatology and Primate Center, Institute of Cognitive Sciences and Technologies, National Research Council (CNR), 00197 Rome, Italy; 4 Department of Dynamic and Clinical Psychology, and Health Studies, Sapienza University, 00185 Rome, Italy; michela.decicco@uniroma1.it; 5 Department of Mother and Child Health, Policlinico Umberto I, Sapienza University, 00161 Rome, Italy

**Keywords:** preterm infants, neurodevelopment, Bayley-III, DP-3, parent-report, early assessment, cognitive development, motor development, socio-emotional development

## Abstract

**Highlights:**

What are the main findings?

- The BSID-III and DP-3 identify similar developmental patterns in preterm infants, with motor development highlighted as an area of greater vulnerability.
- The DP-3 tends to classify more children as typically developing, especially in the cognitive and social-emotional domains, compared to the BSID-III.

What is the implication of the main finding?

- The DP-3 may be appropriate for screening at-risk populations.
- Direct assessments like the BSID-III are more adequate for identifying developmental delays, and differences between the tools highlight the need to understand each assessment’s capacities and limitations when interpreting developmental outcomes.

**Abstract:**

**Background:** Preterm birth is associated with increased risk for neurodevelopmental disorders. Although standardized tools such as the Bayley Scales of Infant and Toddler Development—Third Edition (BSID-III) are widely used for early developmental assessment, parent-report measures may offer complementary and cost-effective alternatives. The Developmental Profile 3 (DP-3) is a parent questionnaire with potential utility in preterm follow-up programs. **Objective:** To compare developmental outcomes of preterm infants at 12 months corrected age assessed using the BSID-III and the DP-3 questionnaires and to evaluate the agreement between these tools across cognitive, language-communication, motor, and social-emotional domains. **Methods:** Fifty-five preterm infants (mean GA = 30.3 weeks; mean BW = 1388 g) were assessed using both the BSID-III (administered by professionals) and the DP-3 (completed by parents) at 12 months corrected age. Mean scores were computed for each domain, and infants were assigned to the corresponding descriptive categories. The agreement between BSID-III and DP-3 scores was statistically evaluated. **Results:** Both instruments identified similar developmental trends, with motor development emerging as the most vulnerable domain for preterm infants. DP-3 scores were higher than BSID-III scores in virtually all domains, and absolute intraclass correlation coefficients showed a generally moderate agreement between measurements. The BSID-III identified significantly more infants at risk in the cognitive and social-emotional areas compared to the DP-3. **Conclusions:** The DP-3 showed fair convergence with the BSID-III, supporting its use as a complementary tool in preterm follow-up. Extending follow-up assessments into later developmental stages will be essential to more accurately determine the predictive validity of the DP-3.

## 1. Introduction

Globally, preterm birth remains a significant public health concern, affecting approximately 10% of all live births [1]. In Italy, prematurity affects about 7% of births, with approximately 0.9% occurring before 32 weeks’ gestation, an extremely vulnerable group at high risk for adverse neurodevelopmental outcomes [2]. This rate has shown a gradual increase over recent decades, driven by various factors such as advanced maternal age, exposure to environmental pollutants, smoking, and the widespread use of assisted reproductive technologies, which are often associated with multiple pregnancies [3,4].

Despite advances in neonatal care, the prevalence of neurodevelopmental impairments among very preterm infants remains considerable: recent literature reports that 10–12% of these infants develop primary neurological or psychiatric conditions, including cerebral palsy, epilepsy, sensory deficits, intellectual disability, or autism spectrum disorder [5,6,7]. Moreover, many show subtler deficits in cognitive, motor, and socio-emotional domains, such as Developmental Coordination Disorder, Specific Learning Disorders, or ADHD [8,9,10].

Performing early neurodevelopmental assessments for preterm infants is highly recommended to identify any delays or atypical developmental patterns as soon as possible [11]. Early detection facilitates timely intervention with support and rehabilitation programs, which is especially effective during the first 12 months of corrected age. This period represents a sensitive window for targeted support while foundational neural pathways are rapidly forming [12].

Among the tools available, the Bayley Scales of Infant and Toddler Development—Third Edition (BSID-III) [13] are the gold standard for early developmental screening and follow-up in preterm populations. These scales have demonstrated robust predictive validity for later cognitive outcomes and are frequently employed in clinical practice and research settings [14].

However, most experts agree on the importance of using multiple sources and methods in developmental assessment, particularly when evaluating at-risk populations such as preterm infants [15,16]. An approach that combines standardized, direct assessments with parent-reported questionnaires is widely suggested, as it enhances diagnostic sensitivity and offers a more comprehensive knowledge of a child’s functioning across various settings. Parent-report instruments represent a cost-effective, time-efficient option for early detection of developmental concerns that may not emerge in structured testing environments [17,18]. They are especially useful in large-scale developmental screening programs and/or low- and middle-income countries, where direct examination of all children may not be feasible due to shortages of trained professionals or logistical barriers in preterm follow-up programs [19]. Parent-report tools have proved to be effective and easy to administer, making them suitable for incorporation into public health plans and early intervention systems [20], allowing timely triage for further evaluation when needed. Indeed, parents are uniquely positioned to observe their child’s everyday functioning and are often the first to notice developmental alterations [21], which have been reported to be strong predictors of later developmental and learning difficulties [22].

Several parent-report instruments have been validated and compared to standardized tests such as the BSID-III, including the PARCA-R (Parent Report of Children’s Abilities–Revised), the ASQ-3 (Ages and Stages Questionnaire—Third Edition), and the Vineland Adaptive Behavior Scales–II (VABS-II). Notably, the PARCA-R and ASQ-3 have been culturally adapted and validated in the Italian context [23,24]. These tools have demonstrated varying degrees of agreement with direct assessments, highlighting both their utility and limitations in detecting developmental concerns in preterm and full-term infants.

Another parent-report instrument widely used in both clinical and research contexts is the Developmental Profile 3 (DP-3). This tool provides a structured overview of a child’s developmental profile across five key developmental domains: Motor, Adaptive Behavior, Social-Emotional, Cognitive, and Communication [25]. Various studies demonstrated the DP-3’s usefulness to evaluate motor skills, global development, and adaptive behaviors in children with diverse neurodevelopmental disorders, including autism spectrum disorder, Down syndrome, and cerebral palsy [26,27,28,29]. However, no published studies have yet used the DP-3 to assess developmental outcomes in preterm infants, nor compared the DP-3 with the BSID-III in preterm or full-term infants. Thus, the present study aims to address this gap by examining developmental profiles of preterm infants at 12 months corrected age using the BSID-III and the DP-3, while comparing their agreement across key domains. To ensure that this comparison reflects differences typically observed in the preterm population, rather than being driven by extreme developmental impairments, we excluded infants with severe neurological or genetic conditions.

## 2. Materials and Methods

### 2.1. Participants

Participants were enrolled through the preterm follow-up programs of Sant’Eugenio and Policlinico Umberto I Hospitals in Rome, Italy from the beginning of 2021 to the end of 2022. Informed consent was obtained from all parents or legal guardians prior to participation. Children were excluded if they presented genetic syndromes, severe neurosensory or motor impairments, or major brain injuries identified via neonatal cranial ultrasound, specifically grade III-IV intraventricular hemorrhage or cystic periventricular leukomalacia.

### 2.2. Instruments

#### 2.2.1. Bayley Scales of Infant and Toddler Development—Third Edition (Bayley, 2006 [13])

The Bayley Scales of Infant and Toddler Development—Third Edition (BSID-III) comprises five scales: Cognitive (Cog), Language (Lang), and Motor (Mot) scales, which are administered through direct interaction with the child, and Adaptive Behavior (GAC) and Social-Emotional (SE) scales, which are based on parent or caregiver reports via standardized questionnaires [13,30]. The Language and Motor scales are further divided into two subscales: Receptive Communication (RC) and Expressive Communication (EC), and Fine Motor (FM) and Gross Motor (GM), respectively. The present study utilized the Cognitive, Language, Motor, and Social-Emotional scales, each providing a composite score (mean = 100, standard deviation (SD) = 15) reflecting the child’s performance relative to age-based norms. To aid in the qualitative interpretation of developmental strengths and potential delays, each numerical score can also be assigned to a descriptive category, such as mean (scores between 85 and 115), below mean (<85), or above mean (>115), based on standard score ranges.

#### 2.2.2. Developmental Profile 3 (DP-3) Questionnaire (Alpern, 2007 [25])

The DP-3 questionnaire is a standardized instrument designed to be administered to parents/caregivers of children aged from 0 to 12 years and 11 months. It includes five scales: Motor, Adaptive Behavior, Social-Emotional, Cognitive, and Communication. For the present study, only the Motor, Social-Emotional, Cognitive, and Communication scales were considered, as these domains are directly comparable to the corresponding Motor, Social-Emotional, Cognitive, and Language scales of the BSID-III [25,31]. The DP-3’s standard scores (M = 100; SD = 15) were derived from raw scores, interpreted according to established normative ranges and categorized into descriptive levels of developmental functioning. Scores above 130 indicate significantly above-mean development, scores between 116 and 130 reflect above-mean development, scores from 85 to 115 are considered mean, scores between 70 and 84 indicate below-mean development, and scores below 70 suggest a developmental delay.

### 2.3. Procedures

Developmental assessments were conducted during the scheduled 12-month corrected age visit. Informed consent was acquired from the parents or legal guardians, who completed the DP-3 questionnaire during the same session. The study was approved by the Ethics Committee of the Azienda Ospedaliero-Universitaria Policlinico Umberto I, Rome (reference number 7471, Prot. 0081/2024).

### 2.4. Statistical Analysis

For each scale (Cognitive, Language-Communication, Motor, and Social-Emotional), we computed means and standard deviations for BSID-III composite scores and DP-3 standard scores and compared them using paired *t*-tests, given the normal distribution of repeated measurements from the same individuals. For each scale, we also computed absolute intraclass correlation coefficients on BSID-III composite scores and DP-3 standard scores to quantify the degree of agreement and consistency between these instruments. Then, we carried out a 2 × 2 chi-square test to compare the number of infants categorized as showing typical development (i.e., assigned to the descriptive categories “mean” and “above mean” scores) versus those classified as at-risk or with delay (i.e., assigned to the descriptive categories “below mean” and “delay” scores) across equivalent domains of the BSID-III and DP-3 questionnaire. The 2 × 2 chi square test was meant to assess whether the observed frequencies of infants falling into each category (typical development vs. at risk/with delay) differed between the two instruments. Statistical analyses were carried out using SPSS Statistics (IBM), version 24.0 and Stata 17.0. A *p*-value < 0.05 (two-tailed) was considered statistically significant.

## 3. Results

### Comparison Between BSID-III and DP-3

The sample consisted of 55 preterm infants (49% female), with a mean chronological age of 14.54 months (SD = 0.76) and a mean corrected age of 12.33 months (SD = 0.67). Table 1 reports infants’ characteristics collected from medical records.

We evaluated whether BSID-III and DP-3 provided an equivalent assessment of child development by reporting descriptive measures for BSID-III composite scores and DP-3 standard scores, and—for each scale—comparing the scores by means of paired *t*-tests and absolute intraclass correlation coefficients. As shown in Table 2, BSID-III composite scores were within the mean range but generally below 90, with the Motor score notably lower, falling below 85 (i.e., more than 1 SD below the normative mean). The DP-3 scores followed a comparable pattern: while mean scores across most scales were slightly higher—typically above 90—the Motor score again showed a marked reduction, with a mean score below 85. This parallel trend across both assessment tools highlights a consistent relative weakness in motor development within this preterm group. With the exception of the Motor domain, DP-3 scores were higher than BSID-III scores in all domains, with this difference reaching significance for the Cognitive and Language-Communication domains (Table 2).

**Figure 1:**
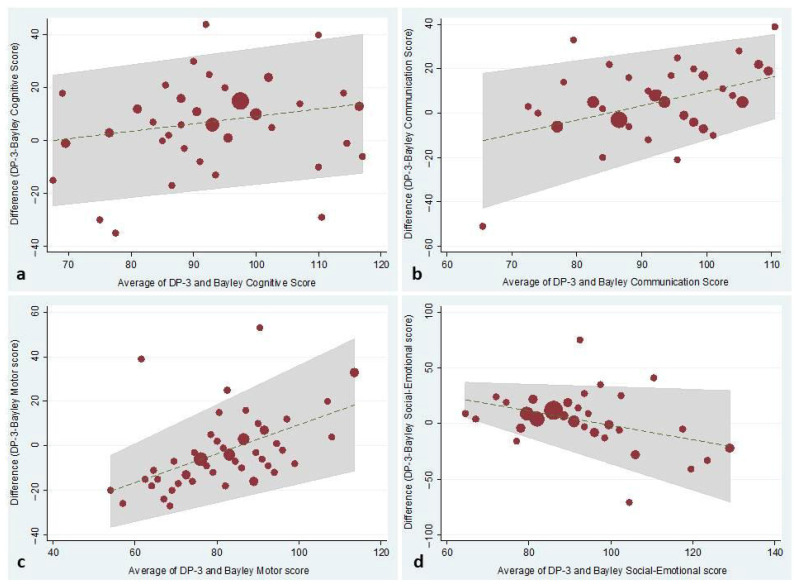
The figure depicts the Tukey mean-difference plots for each developmental domain.

Absolute intraclass correlation coefficients between composite scores of the BSID-III and standard scores of the DP-3 questionnaire were fair for the Cognitive (0.584), Language-Communication (0.502) and Social-Emotional (0.427) scales and good for the Motor scale (0.619). Figure 1 depicts the Tukey mean-difference plots for each developmental domain.

We also assessed whether BSID-III and DP-3 similarly identified infants categorized as showing typical development versus those classified as at-risk or with delay. Table 3 presents a comparison of the number of infants classified as having typical development (i.e., scores at or above the mean) versus those identified as at risk or delayed (i.e., scores below the mean or in the delayed range) across corresponding scales of the BSID-III and DP-3. Statistically significant differences were found in the Cognitive and Social-Emotional domains (see Table 3).

When comparing the number of infants categorized by BSID-III and DP-3 as showing typical development versus those classified as at-risk or with delay, some discrepancies emerged. On the Cognitive scale, the DP-3 identified a significantly higher number of infants as showing typical development (47 infants; 85.5%) compared to the BSID-III (34 infants; 61.8%), while the BSID-III identified more at-risk or delayed infants (DP-3 = 8 infants, 14.5%; BSID-III = 21 infants, 38.2%), χ^2^ = 7.91, *p* = 0.005. On the Language-Communication scale, no significant difference was found between the two instruments (typical developing infants: BSID-III = 41 infants, 74.5%; DP-3 = 48 infants, 87.3%; at-risk/delayed: BSID-III = 14 infants, 25.5%; DP-3 = 7 infants, 12.7%), χ^2^ = 2.88, *p* = 0.089. Similarly, on the Motor scale, percentages were comparable (typical development: BSID-III = 19 infants, 34.5%; DP-3 = 23 infants, 41.8%; at-risk/delayed: BSID-III = 36 infants, 65.5%; DP-3 = 32 infants, 58.2%), χ^2^ = 0.62, *p* = 0.432. As for the Cognitive scale, a significant discrepancy emerged for the Social-Emotional scale: the DP-3 identified a significantly higher number of infants as showing typical development (38 infants; 69.1%) compared to the BSID-III (21 infants; 38.2%), while the BSID-III identified more at-risk or delayed infants (34 infants, 61.8%) than the DP-3 (17 infants, 30.9%), χ^2^ = 15.35, *p* < 0.001.

## 4. Discussion

This study compared two widely used instruments, the BSID-III and the DP-3, for assessing developmental outcomes in preterm infants across four core domains: Motor, Cognitive, Social-Emotional, and Language-Communication. We investigated their agreement at 12 months corrected age and assessed whether they could be used complementarily or interchangeably for developmental screening and follow-up in at-risk populations.

Assessment at 12 months corrected age is a pivotal milestone in the neurodevelopmental follow-up of preterm infants, as this period is particularly sensitive for detecting early deviations from typical development and provides insights into the child’s emerging developmental trajectory [32]. Early identification of subtle vulnerabilities allows for timely, targeted interventions and the formulation of individualized care strategies, which are critical for improving long-term outcomes in this high-risk group [12,33]. Moreover, utilizing corrected age in developmental assessments is essential, as it provides a more accurate reflection of the infant’s developmental status, thereby preventing the over-identification of delays that might occur if chronological age was used instead [34,35].

Our results showed that, in the direct evaluation by means of the BSID-III, Cognitive, Language-Communication, and Social-Emotional scores in preterm infants generally fell within the normative range, although they remained consistently below the standardized mean of 90. This pattern indicates mild developmental lags that may not reach clinical thresholds but nonetheless warrant careful monitoring. These findings are consistent with prior research documenting subtle delays in cognitive and language development among preterm infants. Longitudinal studies have shown that children born very preterm or with very low birth weight (VLBW) frequently perform below their full-term peers on cognitive assessments, with these difficulties often persisting into school age [8,36]. Early language difficulties, even when mild, have been associated with poorer academic and social outcomes, underscoring the importance of proactive monitoring [37]. In this context, identifying mild cognitive and language-communication and social-emotional problems should inform individualized follow-up plans. Ongoing developmental surveillance allows for early intervention and support tailored to each child’s specific needs, which is essential for optimizing long-term outcomes in this vulnerable population.

Additionally, preterm infants, especially those born extremely preterm, show significant delays in the Socio-Emotional domain, including difficulties in self-regulation and intentional use of emotions [38]. Contributing factors may include neurological immaturity, prolonged hospitalization, and reduced early parent–infant interactions. Caregiver mental health also plays a critical role: maternal anxiety or depression can affect both child outcomes and the caregiver’s perception of the child, potentially leading to biased reporting. Gray et al. (2018) highlighted how maternal mental health, motor development, and externalizing behaviors can shape socio-emotional outcomes [39]. Moreover, Jaekel and Aubert (2024) found that environmental and sociolinguistic factors, such as the linguistic distance between a family’s native language and the host country’s language, were linked to greater behavioral and socio-emotional difficulties in very preterm children at age five [40]. Thus, an evaluation relying solely on parent-report measures may provide an incomplete view of socio-emotional functioning. Integrating observational tools and caregiver mental health assessments can offer better knowledge and allow targeted early interventions.

Motor development emerged as the most consistently affected domain across both assessment tools. In our sample, the BSID-III and DP-3 yielded mean motor scores more than one standard deviation below the normative mean. This shared pattern reinforces the well-established vulnerability of motor development in preterm populations [41,42]. Various studies found that children born very preterm or VLBW exhibit significantly poorer motor outcomes, often extending into later childhood and adolescence. Early motor delays may impact later cognitive–motor coordination and autonomy, underscoring the importance of prompt identification and targeted support. The consistent findings across tools regarding motor difficulties strengthen the case for incorporating motor assessments as a core component of routine developmental follow-up in preterm infants. This is particularly important given that early motor delays may also signal broader neurodevelopmental risks.

When directly comparing DP-3 and BSID-III, the present study highlights key differences between instruments across most of the examined developmental domains. First of all, with the only exception of the Motor domain, DP-3 scores were higher than BSID-III scores in all domains, with this difference reaching significance for the Cognitive and Language-Communication domains. Additionally, absolute intraclass correlation coefficients between BSID-III composite scores and DP-3 standard scores showed a generally moderate agreement between measurements, with slightly better results for the Motor domain. Some discrepancies between BSID-III and DP-3 emerged also when comparing the number of children categorized by the two instruments as showing typical development versus those classified as at-risk or with delay. In the Cognitive and, especially, Socio-Emotional domains, the BSID-III identified a significantly higher proportion of children with developmental difficulties than the DP-3. These differences likely stem from the distinct methods used by the two instruments.

As for the Cognitive domain, the BSID-III involves structured, clinician-administered tasks that directly evaluate cognitive functions, allowing for the detection of subtle developmental delays. For instance, the BSID-III contains specific tasks for 12-month-old children, such as retrieving hidden objects, manipulating toys in various ways, or putting a cube into a cup on order. These tasks are administered under controlled conditions, allowing a more objective assessment. Conversely, the DP-3 Cognitive scale asks caregivers whether the child engages in similar behaviors, such as searching for hidden toys, showing interest in new objects, or responding to joint attention cues (e.g., looking where someone is pointing). Although these items address overlapping competencies, they depend on the caregiver’s interpretation and may not capture subtler difficulties. Caregiver’s reports, while valuable, may be influenced by parental perception and subjectivity, potentially leading to an overestimation of typical development. However, it is essential to note that clinician-administered measures such as the BSID-III also have limitations. Recent evidence suggests that parental and medical classifications of neurodevelopment can differ substantially, not only due to parental overestimation but also because standardized assessments may not fully capture the child’s abilities and real-life functioning [43]. Therefore, discrepancies between caregiver reports and BSID-III scores should be interpreted cautiously, considering the subjectivity of caregiver assessments and the potential constraints of standardized tools.

In the Language domain, no significant difference was observed between the measurements obtained by the two tools, indicating that the DP-3 and the BSID-III provide comparable language development assessments at 12 months corrected age. This result likely reflects that early language milestones, such as babbling, vocalizations, and simple word use, are relatively easy for caregivers to observe and report. Moreover, despite the lack of statistical significance, the BSID-III identified a higher proportion of children with language difficulties (25.5%) than the DP-3 (12.7%). This suggests that while parent-report tools are helpful, a direct approach may be more sensitive in detecting subtle language delays in preterm infants. Consequently, supplementing caregiver reports with direct clinical assessments remains important for a comprehensive evaluation.

As for the Social-Emotional domain, for which the highest discrepancy between instruments emerged, this may be due to how these tools identify socio-emotional difficulties. The BSID-III may be more sensitive to early self-regulation and social communication challenges, assessing subtle behaviors such as joint attention, emotional responsiveness, and gestures to express needs. For example, at 12 months, the BSID-III Social-Emotional scale includes items such as “Does your child show that he/she understands your actions or gestures by responding appropriately?” (e.g., looking at something you point to, smiling in response to positive affect, or pausing when said “no” in a firm voice) and “Does your child engage in a sequence of actions to express wants or participate in play?” (e.g., smiling, leaning in for a hug, then taking your hat and placing it on their head with pride) [13]. In contrast, the DP-3 Social-Emotional scale focuses on broader, more observable behaviors, such as welcoming familiar adults or showing interest in other children’s play. Examples of the DP-3 assessed behaviors are “Is he/she interested in objects or games that other children like?” and “Does he/she greet familiar adults by hugging them or making a sound that means ‘hello’?” [25]. These items reflect socially appropriate behaviors and interests in others. However, they may not fully capture the depth of socio-emotional regulation, and they may have a limited ability to detect milder emotional regulation or interactive signaling impairments during infancy.

Finally, for the Motor domain, the results obtained by the BSID-III and DP-3 were comparable, with both tools effectively identifying motor delays in preterm infants. However, a slightly higher proportion of children being identified with motor delays on the BSID-III may reflect its more structured and direct observation of motor behaviors, which could be more sensitive to motor deficits that are not easily captured through parent reports. Motor development in preterm infants is often complex, and clinical assessments like the BSID-III are better equipped to detect even subtle motor delays that might be overlooked by parents.

In conclusion, this study suggests that the BSID-III and DP-3 highlight similar developmental patterns in preterm infants, particularly pointing to motor development as an area of greater vulnerability. Nonetheless, the agreement between measures obtained by these instruments appears overall moderate, and DP-3 classifies a larger proportion of children as typically developing, especially in cognitive and social-emotional domains. These differences likely reflect variations in assessment methods or in the specific content explored within the same modality. An integrated approach that includes standardized assessments and parent-report measures can provide a more exhaustive understanding of a child’s development, as each method offers unique and complementary information. In large-scale study or in settings where direct assessment is not feasible, the DP-3 may serve as a practical alternative, as long as its limitations and differences from clinician-administered developmental scales are acknowledged.

Limitations of this study include a relatively small sample size and the assessment at a single time point, which constrain generalizability and understanding of long-term outcomes. Thus, future studies are warranted to extend follow-up assessments to later developmental stages in order to evaluate the long-term agreement between these tools. In this perspective, longitudinal studies would contribute to refining the best practices for early identification and intervention in the preterm population.

## Figures and Tables

**Table 1:** Group characteristics.

Preterm Infants (N= 55)	
Female N (%)	27 (49%)
Corrected age (months) M ± SD [range]	12.33 ± 0.67 [12–14]
Chronological age (months) M ± SD [range]	14.54 ± 0.76 [13–16]
GA (weeks) M ± SD [range]	30.31 ± 2.12 [26–34]
BW (grams) M ± SD [range] Apgar score at 5’ M ± SD [range]	1388.1 ± 438.7 [629–2348] 8.3 + 0.9 [7–10]
Multiple births N (%)	21 (38.0)
Hospitalization in NICU (days) M ± SD [range]	50.8 ± 20.7 [10–117]
Maternal age (years) M ± SD [range]	34.7 ± 7.5 [18–52]

Note: N = number; M = mean; SD = standard deviation; GA = gestational age at birth; BW = birth weight; NICU = neonatal intensive care unit.

**Table 2:** For each developmental domain, Bayley-III composite scores, DP-3 questionnaire standard scores (mean ± SD [range]) and paired *t*-tests (N = 55).

	BSID-III Composite Scores	DP-3 * Standard Scores	*t* (df)	*p*
Cognitive	89.5 ± 12.9 [60–125]	96.8 ± 15.9 [60–130]	**3.562 (54)**	**0.001**
Language	89.8 ± 9.6 [63–106]	94.6 ± 15.4 [40–130]	**2.423 (54)**	**0.019**
Motor	83.2 ± 11.3 [42–106]	81.0 ± 18.9 [44–130]	−0.978 (54)	0.332
Social-Emotional	89.3 ± 21.09 [55–140]	93.3 ± 13.6 [69–131]	1.401 (54)	0.167

Note: composite scores (M = 100; SD = 15); standard scores (M = 100; SD = 15). * DP-3 scales reordered to match BSID-III for comparison. Significant differences are evidenced in bold. In Cognitive and Language-Communication domains, DP-3 scores were significantly higher than BSID-III scores.

**Table 3:** Distribution of infants (N = 55) across descriptive categories in BSID-III and DP-3 and comparison of the number of infants categorized as showing typical development (i.e., mean or above-mean scores) versus those classified as at-risk or with delay (i.e., below-mean or delay scores) across equivalent scales of the BSID-III and DP-3 (Chi-square; df = 1). Significant differences are evidenced in bold.

Scale	Descriptive Categories N (%)					Typical Development	At Risk/ with Delay	Chi Square	*p*
	Much Above Mean	Above Mean	Mean	Below Mean	Delay	Much Above Mean/ Above Mean/Mean	Below Mean/ Delay		
Cognitive BSID-III		2 (3.6)	32 (58.2)	18 (32.7)	3 (5.5)	34 (61.8)	21 (38.2)	**7.91**	**0.005**
Cognitive DP-3		5 (9.1)	42 (76.4)	3 (5.4)	5 (9.1)	47 (85.5)	8 (14.5)		
Language BSID-III			41 (74.5)	11 (20.0)	3 (5.5)	41 (74.5)	14 (25.5)	2.88	0.089
Communication DP-3		6 (10.9)	42 (76.4)	6 (10.9)	1 (1.8)	48 (87.3)	7 (12.7)		
Motor BSID-III			19 (34.6)	29 (52.7)	7 (12.7)	19 (34.5)	36 (65.5)	0.62	0.432
Motor DP-3		4 (7.3)	19 (34.5)	18 (32.7)	14 (25.5)	23 (41.8)	32 (58.2)		
Social-Emotional BSID-III		8 (14.6)	13 (23.6)	27 (49.1)	7 (12.7)	21 (38.2)	34 (61.8)	**10.57**	**0.001**
Social-Emotional DP-3	1 (1.8)	3 (5.5)	34 (61.8)	13 (23.6)	4 (7.3)	38 (69.1)	17 (30.9)		

Note: Much Above Mean > 130; Above Mean = 116–130; Mean = 85–115; Below Mean = 70–84; Delay = < 70. Along both the Cognitive and Social-Emotional scales, the DP-3 identified a higher proportion of infants as showing typical development compared to the BSID-III, while the BSID-III identified more infants at risk or with delay.

## Data Availability

The data that support the findings of this study are available from the corresponding author upon reasonable request.

## Abbreviations

The following abbreviations are used in this manuscript:

BSID-III	Bayley Scales of Infant and Toddler Development—Third Edition
DP-3	Developmental Profile 3
DCD	Developmental coordination disorder
SLD	Specific learning disorders
ADHD	Attention-deficit/hyperactivity disorder
PARCA-R	Parent Report of Children’s Abilities–Revised
ASQ-3	Ages and Stages Questionnaire—Third Edition
VABS-II	Vineland Adaptive Behavior Scales-II
SE	Social-Emotional scale
RC	Receptive communication
EC	Expressive communication
FM	Fine motor
GM	Gross motor
SD	Standard deviation
VLBW	Very low birth weight
